# Identification of Patients With Early Rheumatoid Arthritis: Challenges and Future Directions

**DOI:** 10.1080/17402520600877794

**Published:** 2006

**Authors:** Catalina Orozco, Nancy J. Olsen

**Affiliations:** UT Southwestern Medical Center Dallas TX USA

## Abstract

Rheumatoid arthritis (RA) is the most common form of inflammatory arthritis that affects the adult population. Early diagnosis and treatment are the cornerstones to prevent joint damage and avoid long-term costs and disability. This article reviews the limitations of the currently available tools for the evaluation of patients with early arthritis, including clinical assessment, serologic markers and imaging modalities. It also discusses gene expression analysis, a newer and potentially promising approach to the early diagnosis of RA.


*Clinical & Developmental Immunology*, June–December 2006; 13(2–4): 295–297

Taylor & Francis

Taylor & Francis Group

### Identification of patients with early rheumatoid arthritis: Challenges and future directions

CATALINA OROZCO & NANCY J. OLSEN

*UT Southwestern Medical Center, Dallas, TX, USA*

**Abstract**
Rheumatoid arthritis (RA) is the most common form of inflammatory arthritis that affects the adult population. Early diagnosis and treatment are the cornerstones to prevent joint damage and avoid long-term costs and disability. This article reviews the limitations of the currently available tools for the evaluation of patients with early arthritis, including clinical assessment, serologic markers and imaging modalities. It also discusses gene expression analysis, a newer and potentially promising approach to the early diagnosis of RA.

**Keywords:** *Early rheumatoid arthritis, gene expression analysis, inflammatory arthritis, early synovitis*

### Introduction

Rheumatoid arthritis (RA) is the most common inflammatory arthritis, affecting 0.5–1% of the population. The identification of the early stages of the disease is challenging, as the pathogenesis of RA remains unknown despite extensive research done in the last three decades. To date, there are no specific diagnostic criteria for early RA, hence the diagnosis relies on the 1988 American College of Rheumatology classification criteria for RA (Arnett et al. 1988) and the duration of the arthritic symptoms. These criteria have two caveats: first, they were created to bring uniformity to the subjects studied in clinical research, rather than for diagnostic purposes. Second, they were developed based on the characteristics of patients with long-standing disease, making them insensitive to identify patients with early RA.

### *Importance of the problem*

RA is not only a prevalent disease but it is also very costly. It results in an economic burden of $20 billion/year in the US alone, from both the direct and indirect costs. Moreover, most of the costs come from joint damage, which occurs early in the disease and evolves in a linear and progressive fashion (Fuchs et al. 1989). Around 40% of patients with RA will develop bone erosions as early as 6 months and up to 70% in the first 2 years of onset of symptoms (Hulsmans et al. 2000).

However, early treatment can prevent joint damage and preserve work capacity (Lard et al. 2001; Puolakka et al. 2005) making the need for early identification of patients imperative, and the institution of early aggressive treatment to suppress inflammation and ultimately prevent joint destruction crucial.

### *So, why not to treat all patients with early polyarthritis as rheumatoid arthritis?*

Although this seems an attractive approach, there are several issues inherent to the disease itself and its treatment. The first problem is that patients presenting with early synovitis are very heterogeneous. In the study of subjects with symptoms for less than 6 months, different cohorts have shown that at the time of the first evaluation, the prevalence of RA is very broad (25–60%) (Hitchon et al. 2005), and 40–75% of patients met criteria for other diagnoses such as psoriatic arthritis, spondyloarthropathies or undifferentiated polyarthritis (UP). To add to the confusion, a subset (15–50%) of patients initially classified as UP will develop a classifiable disease like RA during follow up.

Correspondence: C. Orozco, UT Southwestern Medical Center, Dallas, TX, USA. E-mail: catalinaorozco@hotmail.com

ISSN 1740-2522 print/ISSN 1740-2530 online © 2006 Taylor & Francis
DOI: 10.1080/17402520600877794

A second consideration is the fact that the prognosis and treatment of UP are different from those of RA. In general, UP has a more benign course with higher rates of spontaneous remission and with only a minority of patients having persistent disease and erosions during follow up (Morel et al. 2000). This becomes important when one realizes that the treatment of RA is far from being benign and risk-free.

### Available tools for the identification of patients with early RA

As clinicians we rely on three tools to diagnose patients as having RA:

1. Clinical criteria.
2. Serologic markers.
3. Radiographic evidence of joint damage.

As previously discussed, clinical criteria remain an insensitive way to approach patients with early arthritis due to the heterogeneity of the early arthritis population, the lack of validated criteria that are specific for early RA, and the fact that many patients can evolve into a defined diagnostic category over time.

It is also remarkable that despite extensive research we lack a biologic marker to be used as a screening tool in the clinical setting. For many years, rheumatoid factor (RF) has been used as an aid in the diagnosis of RA, and it is one of the items in the ACR classification criteria for RA. Unfortunately, sensitivity and specificity of RF for RA, even in established forms of the disease, are only 80 and 85%, respectively. A second marker that has become widely used in the last few years is antibodies against cyclic citrullinated peptide (CCP). Despite the high specificity of CCP antibodies for RA (96%), they have low sensitivity (60–80%) and therefore are not useful as a screening tool (Avouac et al. 2006).

Imaging modalities such as MRI and ultrasound of hands and feet have attracted considerable interest as possible aids in the identification of patients with early RA. Several publications have demonstrated that these modalities are more useful than conventional X-ray in detecting erosions and synovitis (Hoving et al. 2004). However, there are several concerns with their use including the reproducibility of the findings, as they can be operator/reader dependent, their cost, and the time they demand. Finally, the precise long-term implications of MRI abnormalities and their role in treatment decisions are still not clear.

It is apparent that the need for additional tools to aid in the early diagnosis of RA becomes crucial in order to institute prompt treatment to prevent disability.

### Future directions

Gene expression analysis has emerged as a potentially useful tool in the research and approach to patients with complex diseases, such as autoimmune conditions. This approach is a method of analysing the differential expression of thousands of species of messenger RNA (mRNA) simultaneously in two different samples. This novel technique permits the comparison of the complex changes that occur in cells and tissues of the immune system in health and disease.

Using this technique, accurate classification of patients with connective tissue disease has been possible even when the clinical scenario is not known. For example, utilization of gene array analysis of synovial specimens has shown to be useful in delineating differences between patients with established RA and those with osteoarthritis (Devauchelle et al. 2004). Regrettably, synovial samples are not readily available in clinical practice as their procurement involves an invasive procedure. Furthermore, in the earliest RA patients, synovial tissue is not readily available.

More recently, Olsen et al. (2004) attempted to circumvent the need for using synovial tissue for gene expression analysis by using peripheral blood mononuclear cells. They have shown that a specific signature on gene array in peripheral blood mononuclear cells is present in patients with early RA. This signature differentiates them from patients with established RA and healthy controls, suggesting that it may be possible to profile patients with early arthritis who will meet classification criteria for RA in follow up, and who may benefit from close and perhaps more aggressive treatment. This approach has the potential of providing an easy, readily accessible tool to clinicians in both the primary care setting and in rheumatology clinics to evaluate these patients, with the advantage of not requiring invasive procedures, expensive equipment or trained readers.

Despite the initial enthusiasm generated by these findings, larger studies are needed to validate and standardize these data, and to develop a clinically useful tool to diagnose patients with early RA.

### Conclusion

Early identification of patients with RA is crucial to prevent joint damage and disability. Despite great advance in our understanding of the disease, we still rely on less than ideal tools such as the combination of clinical findings, serologic markers and imaging techniques to identify such patients. New horizons to approach and diagnose patients with early arthritis are opening with the use of newer techniques such as gene expression analysis. However, we should be cautious until larger and well-standardized studies are performed.

### References

Arnett FC, Edworthy SM, Bloch DA, McShane DJ, Fries JF, Cooper NS, Healey LA, Kaplan SR, Liang MH, Luthra HS, et al. 1988. The American Rheumatism Association 1987

revised criteria for the classification of rheumatoid arthritis. Arthritis Rheum 31:315–324.

Avouac J, Gossec L, Dougados M. 2006. Diagnostic and predictive value of anti-CCP (cyclic citrullinated protein) antibodies in rheumatoid arthritis: A systematic literature review. Ann Rheum Dis, 65: 845–851.

Devauchelle V, Marion S, Cagnard N, Mistou S, et al. 2004. DNA microarray allows molecular profiling of rheumatoid arthritis and identification of pathophysiological targets. Genes Immun 5:597–608.

Fuchs HA, Kaye JJ, Callahan LF, Nance EP, Pincus T. 1989. Evidence of significant radiographic damage in rheumatoid arthritis within the first 2 years of disease. J Rheumatol 16:585–591.

Hitchon CA, Peschken CA, Shaikh S, El-Gabalawy HS. 2005. Early undifferentiated arthritis. Rheum Dis Clin North Am 31(4): 605–626.

Hoving JL, Buchbinder R, Hall S, Lawler G, et al. 2004. A comparison of magnetic resonance imaging, sonography, and radiography of the hand in patients with early rheumatoid arthritis. J Rheumatol 31:663–675.

Hulsmans HM, Jacobs JW, van der Heijde DM, van Albada-Kuipers GA, Schenk Y, Bijlsma JW. 2000. The course of radiologic damage during the first six years of rheumatoid arthritis. Arthritis Rheum 43:1927–1940.

Lard LR, Visser H, Speyer I, Vander Horst-Bruinsma IE, et al. 2001. Early versus delayed treatment in patients with recent-onset rheumatoid arthritis: Comparison of two cohorts who received different treatment strategies. Am J Med 111:446–451.

Morel J, Legouffe MC, Bozonat MC, Sany J, et al. 2000. Outcomes in patients with incipient undifferentiated arthritis. Joint Bone Spine 67(1):49–53.

Olsen NJ, Sokka T, Seehorn CL, Kraft B, et al. 2004. A gene expression signature for recent onset rheumatoid arthritis in peripheral blood mononuclear cells. Ann Rheum Dis 63: 1387–1392.

Puolakka K, Kautiainen H, Möttönen T, et al. 2005. Early supression of disease activity is essential for maintenance of work capacity in patients with recent onset rhematoid arthritis. Arthritis Rheum 52:36–41.

